# Supplementary material for: mcRigor: a statistical method to enhance the rigor of metacell partitioning in single-cell data analysis
Source: Nat Commun. 2025 Sep 29;16:8602. doi: 10.1038/s41467-025-63626-5 (PMC12480752; doi:10.1038/s41467-025-63626-5)
Supplement: Supplementary file 1 — Supplementary Information [file 41467_2025_63626_MOESM1_ESM.pdf]

Supplementary Information
mcRigor: a statistical method to enhance the rigor of metacell partitioning in single-cell data analysis

Pan Liu[1] and Jingyi Jessica Li[1,2,3,4,5]

[1]Department of Statistics and Data Science, University of California, Los Angeles, CA, USA
[2]Department of Biostatistics, University of California, Los Angeles, CA, USA.
[3]Department of Computational Medicine, University of California, Los Angeles, CA, USA
[4]Department of Human Genetics, University of California, Los Angeles, CA, USA
[5]Bioinformatics Interdepartmental Ph.D. Program, University of California, Los Angeles, CA, USA

Supplementary Figures

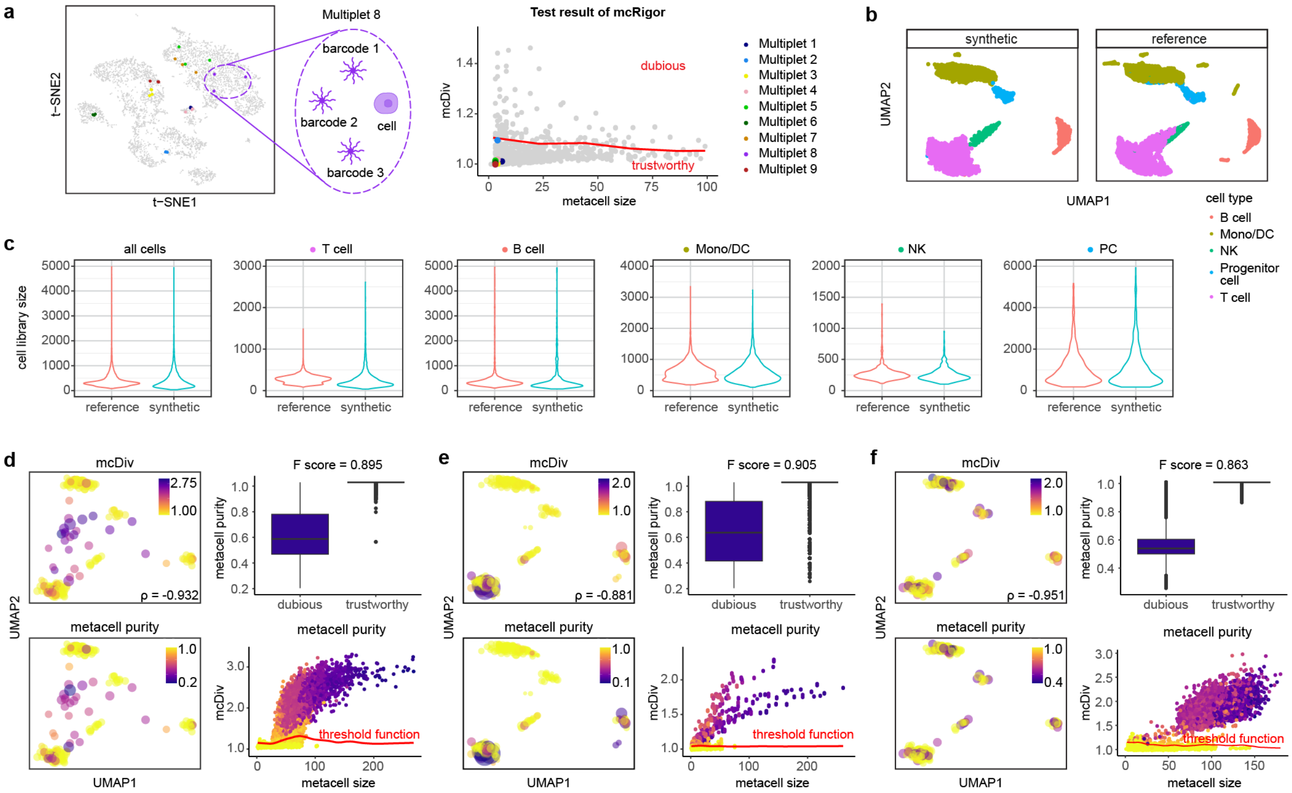

Supplementary Fig 1: Justification of the mcRigor method. a, Barcode multiplets justify the statistical definition of metacell (left) and the reliability of mcRigor (right). **b,** UMAP plots of the semi-synthetic data and the reference real data. **c,** Violin plots of cell library sizes for the semi-synthetic data versus the reference data. **d,** mcRigor effectively measures metacell heterogeneity and detects dubious metacells from the SEACells partition on the semi-synthetic data. Left: UMAP plots of the metacells colored by mcDiv values versus metacell purity (with Pearson correlation $\rho = -0.932$). Top right: mcRigor distinguishes between ground-truth dubious and trustworthy metacells with high accuracy (F-score = 0.895). Bottom left: The scatter plot of mcDiv versus metacell size with the dubious metacell detection threshold function. **e-f,** mcRigor effectively measures metacell heterogeneity and detects dubious metacells from the SuperCell (e) and the MetaQ (f) partition on the semi-synthetic data. Similar to **d**, the Pearson correlation is $\rho = -0.881, -0.951$, and the F-score is $0.905, 0.863$, respectively.

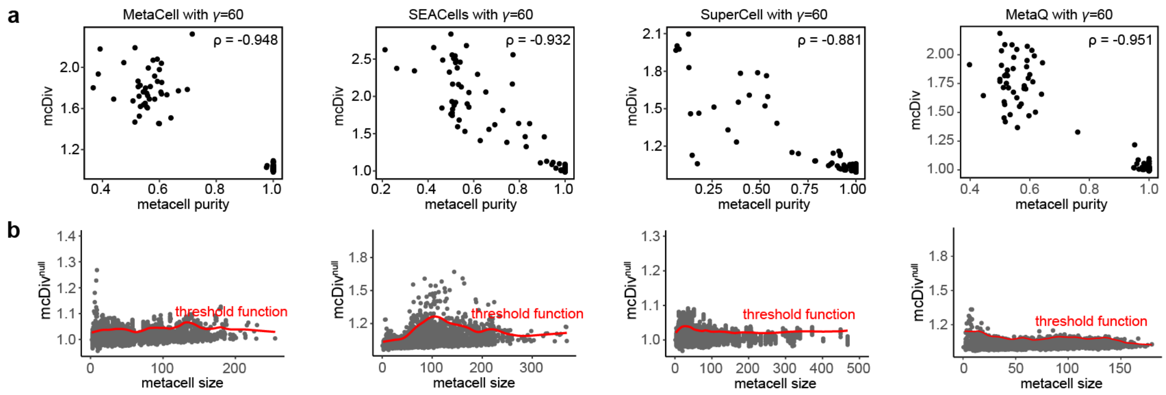

Supplementary Fig 2: Additional simulation results of mcRigor on the semi-synthetic dataset. a, The mcDiv statistic is highly correlated with true metacell purity. ρ stands for Pearson correlation. **b,** Scatter plots showing the distributions of the null statistic value, $\text{mcDiv}^{\text{null}}$, contructed by mcRigor, and the metacell-size-specific threshold function for detecting dubious metacells (as those below their size-specific thresholds).

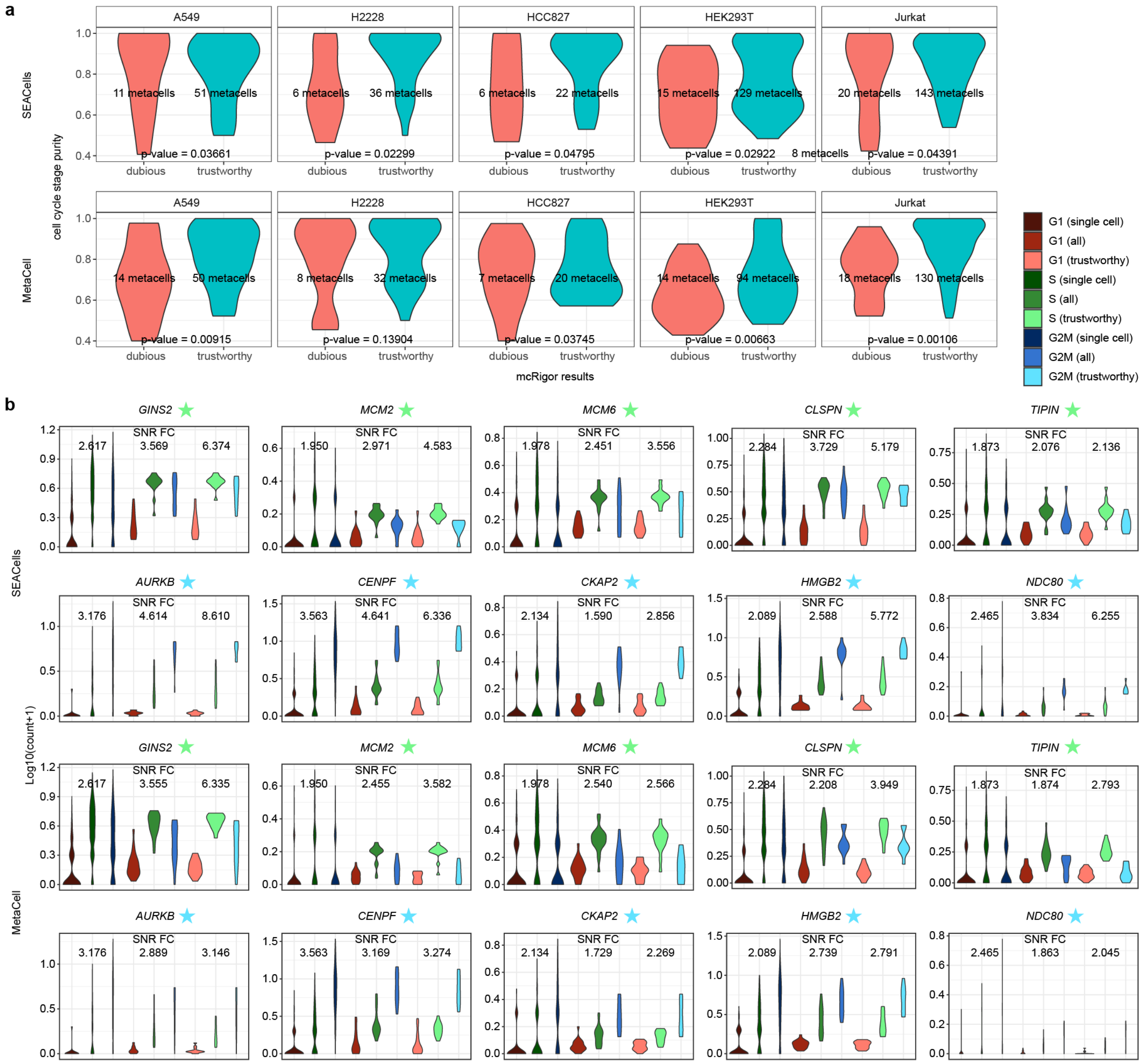

Supplementary Fig 3: mcRigor's trustworthy metacells reveal cell-cycle phases within cell lines. a, Violin plots comparing the cell cycle-phase purity distributions of dubious metacells and trustworthy metacells. Trustworthy metacells consistently exhibit higher purity than dubious metacells. **b,** Violin plots displaying the $\log_{10}$(count+1) expression levels of 10 cell-cycle marker genes across single cells, all metacells ("all"), and trustworthy metacells ("trustworthy"). The metacells were generated by two metacell methods: SEACells and MetaCell, with $\gamma = 20$. SNR FC represents the fold change in signal-to-noise ratio for the phase associated with each marker gene (indicated by a star) relative to the other two phases.

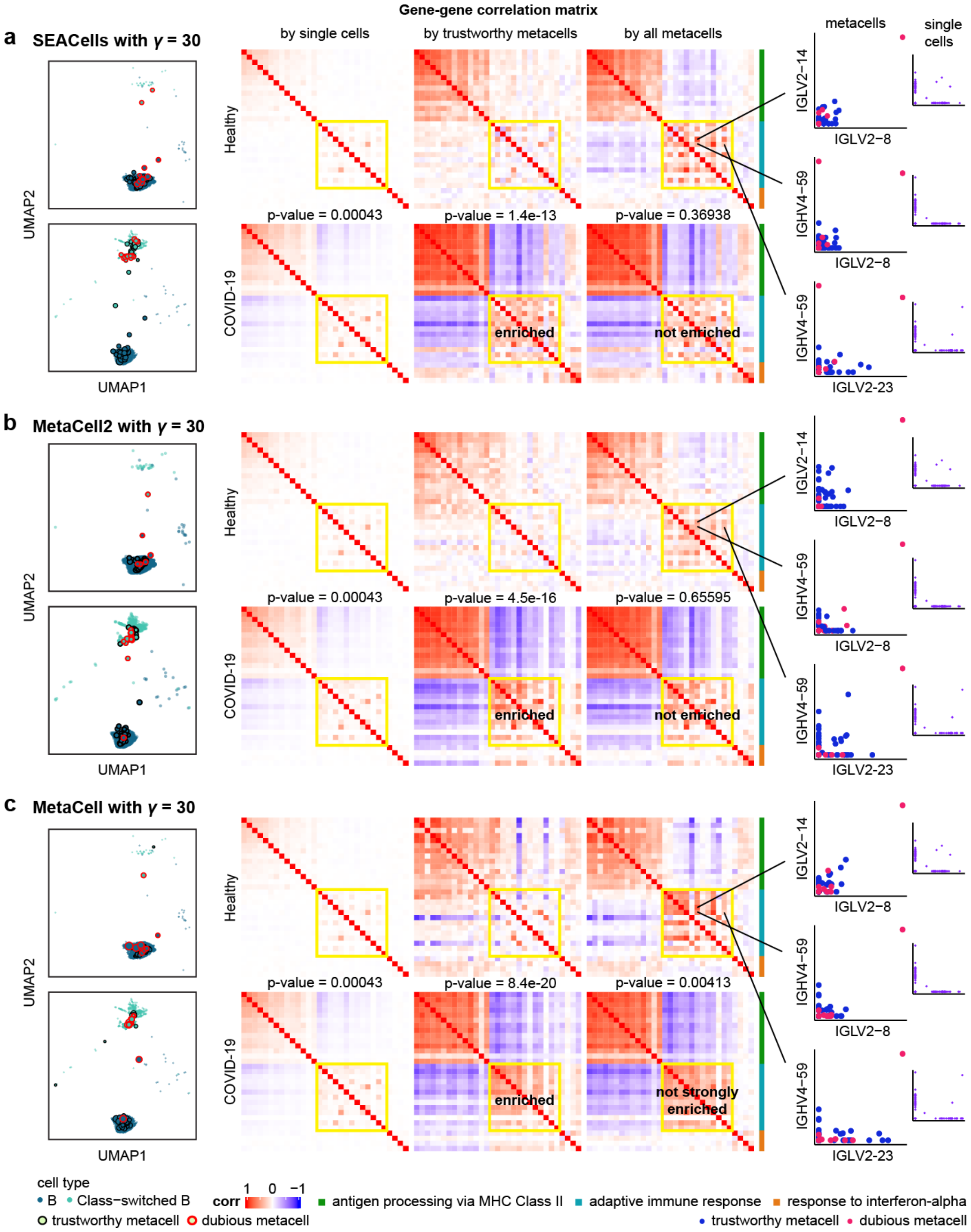

Supplementary Fig 4: mcRigor reveals gene co-expression modules enriched for COVID-19 compared to healthy control by removing signal distortion caused by dubious metacells. This is a continuation of Fig 1e in the main text with analysis results for three more metacell methods: SEACells (**a**), MetaCell2 (**b**), and MetaCell (**c**).

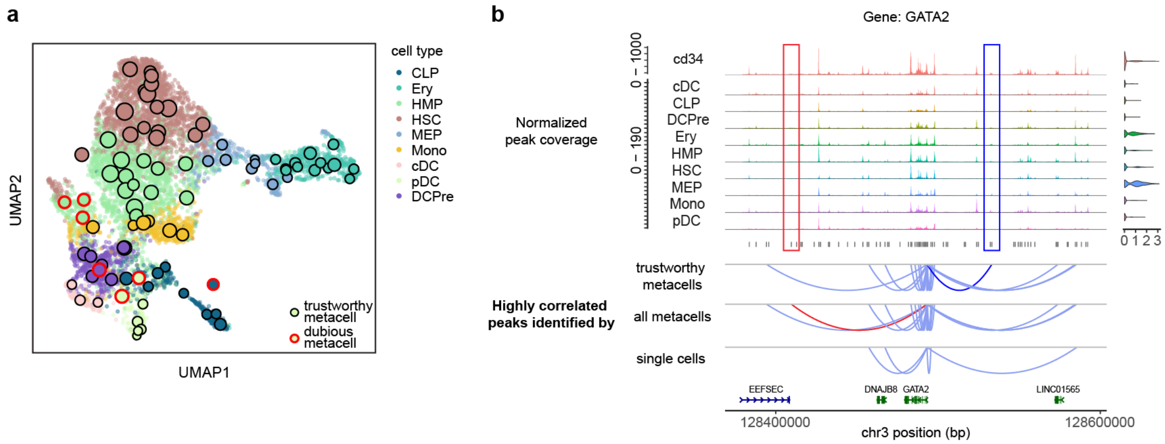

Supplementary Fig 5: mcRigor identifies dubious metacells over the original SEACells partitioning and rectifies gene regulatory inference by removing the dubious metacells. a, UMAP plot showing dubious metacells detected by mcRigor from the original SEACells partitioning. **b,** Highly correlated peaks for gene *GATA2* identified using trustworthy metacells, all metacells, or single cells. This is the complete version for the right panel of Fig 1f (right) in the main text, where we only showed peak signal coverage plots for three cell types (Ery, HSC, and MEP).

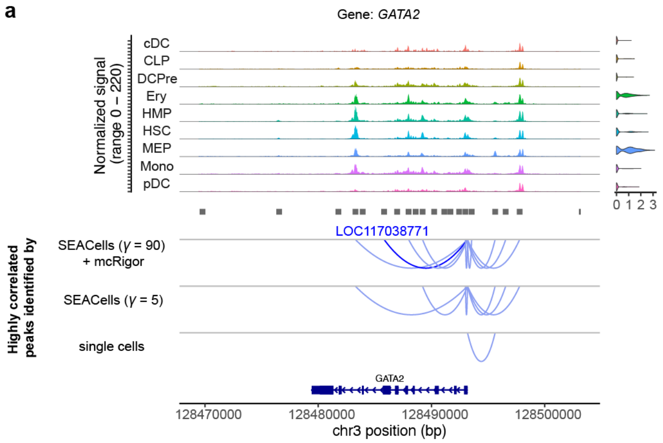

Supplementary Fig 6: mcRigor rectifies gene regulatory inference and gives higher power than performing metacell partitioning under a a sufficiently small granularity level. a, Highly correlated peaks for gene *GATA2* identified using trustworthy metacells at large granularity level (SEACells with $\gamma = 90 +$ mcRigor), all metacells at small granularity level (SEACells with $\gamma = 5$), or single cells. **b,** Highly correlated peaks for gene *TAL1* identified using trustworthy metacells at large granularity level (SEACells with $\gamma = 90 +$ mcRigor), all metacells at small granularity level (SEACells with $\gamma = 5$), or single cells.

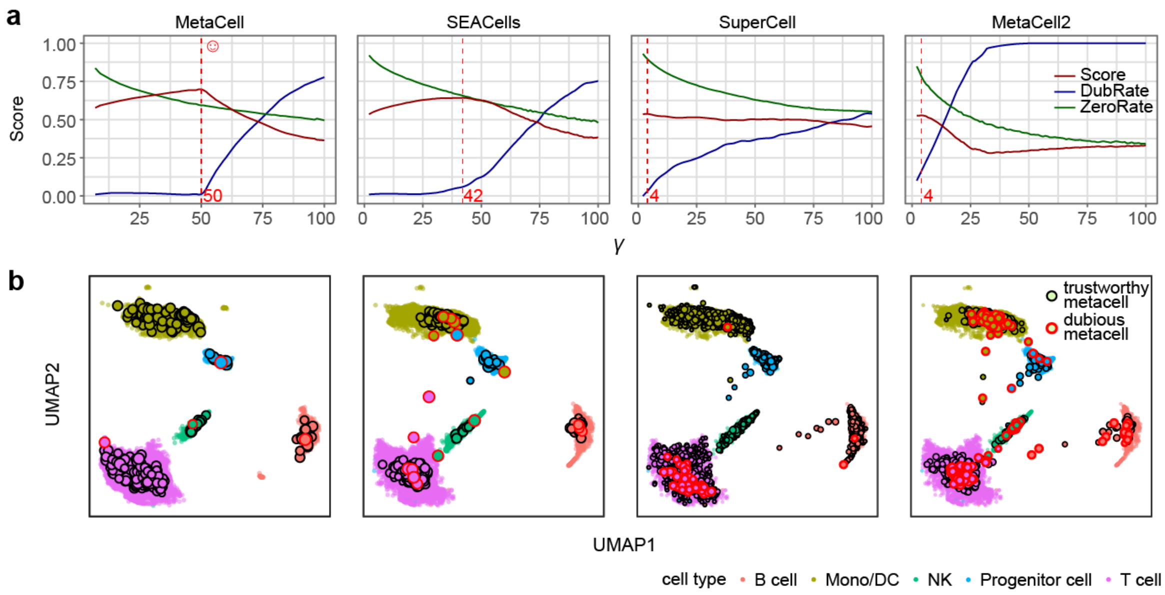

Supplementary Fig 7: mcRigor optimizes metacell partitioning for the semi-synthetic dataset. a, Line plots showing the evaluation scores provided by mcRigor. The vertical red dashed lines mark the optimal γ selected for each metacell method. The red smiling face marks the optimal metacell partition selected across all method-hyperparameter configurations. **b,** Single-cell UMAP plots showing the optimal metacell partitioning for each method, with dubious metacells highlighted in red circles.

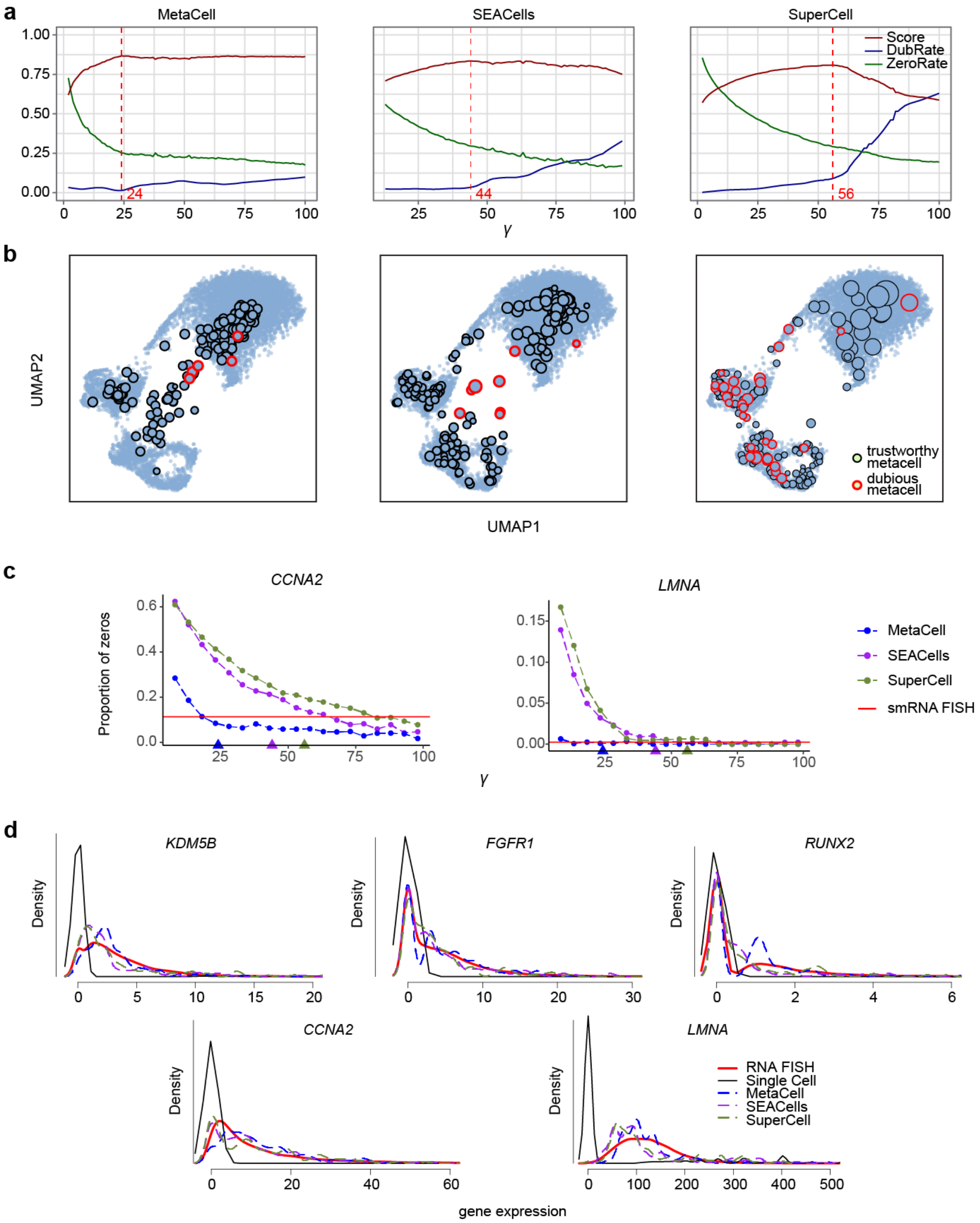

Supplementary Fig 8: Additonal results from applying mcRigor to the Drop-seq + smRNA FISH dataset. a, The evaluation scores provided by mcRigor. The vertical red dashed lines mark the optimal γ selected for each metacell method. **b,** Single-cell UMAP plots showing the optimal metacell partitioning for each method, with dubious metacells highlighted in red circles. **c,** Line plots showing zero proportions for genes *CCNA2* and *LMNA* under metacell partitionings generated by the three metacell methods with varying γ values (with triangles indicating the optimal γ values selected in **a**). The red horizontal line marks the zero proportion in the smRNA FISH data. **d,** Density plots showing gene expression from the single cell profiles, metacell profiles (generated by the optimal γ for each metacell method), and the smRNA FISH profiles.

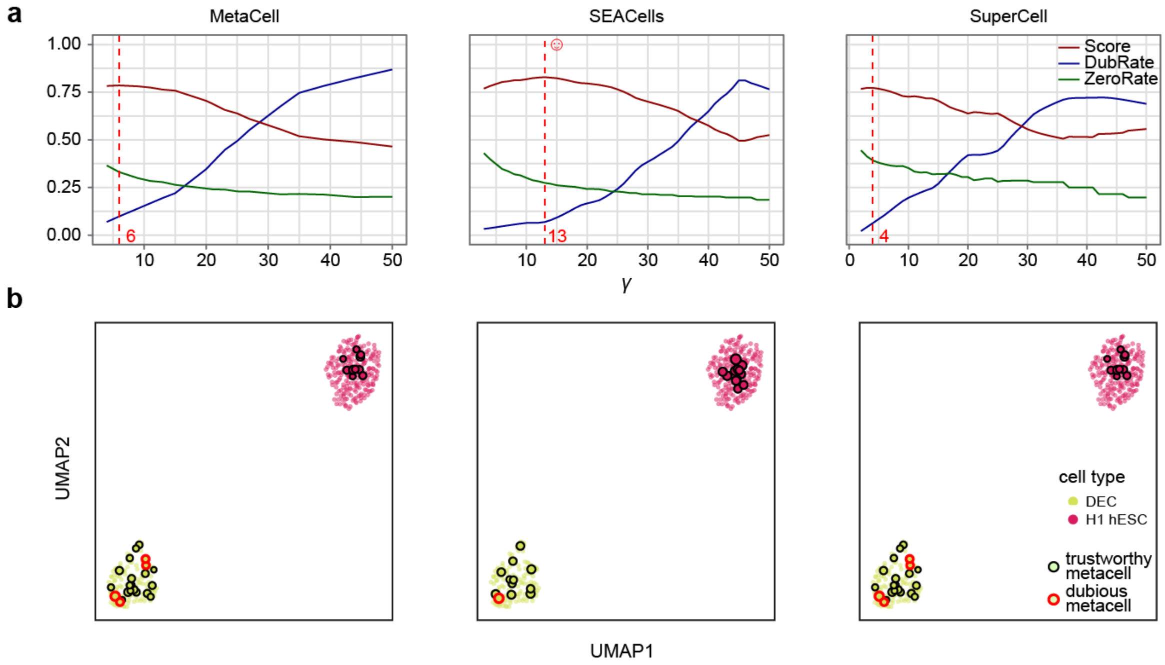

Supplementary Fig 9: Additonal results from applying mcRigor to the scRNA-seq + bulk ESC dataset. a, The evaluation scores provided by mcRigor. The vertical red dashed lines mark the optimal γ selected for each metacell method. The red smiling face marks the optimal metacell partition selected across all method-hyperparameter configurations. **b,** Single-cell UMAP plots showing the optimal metacell partitioning for each method, with dubious metacells highlighted in red circles.

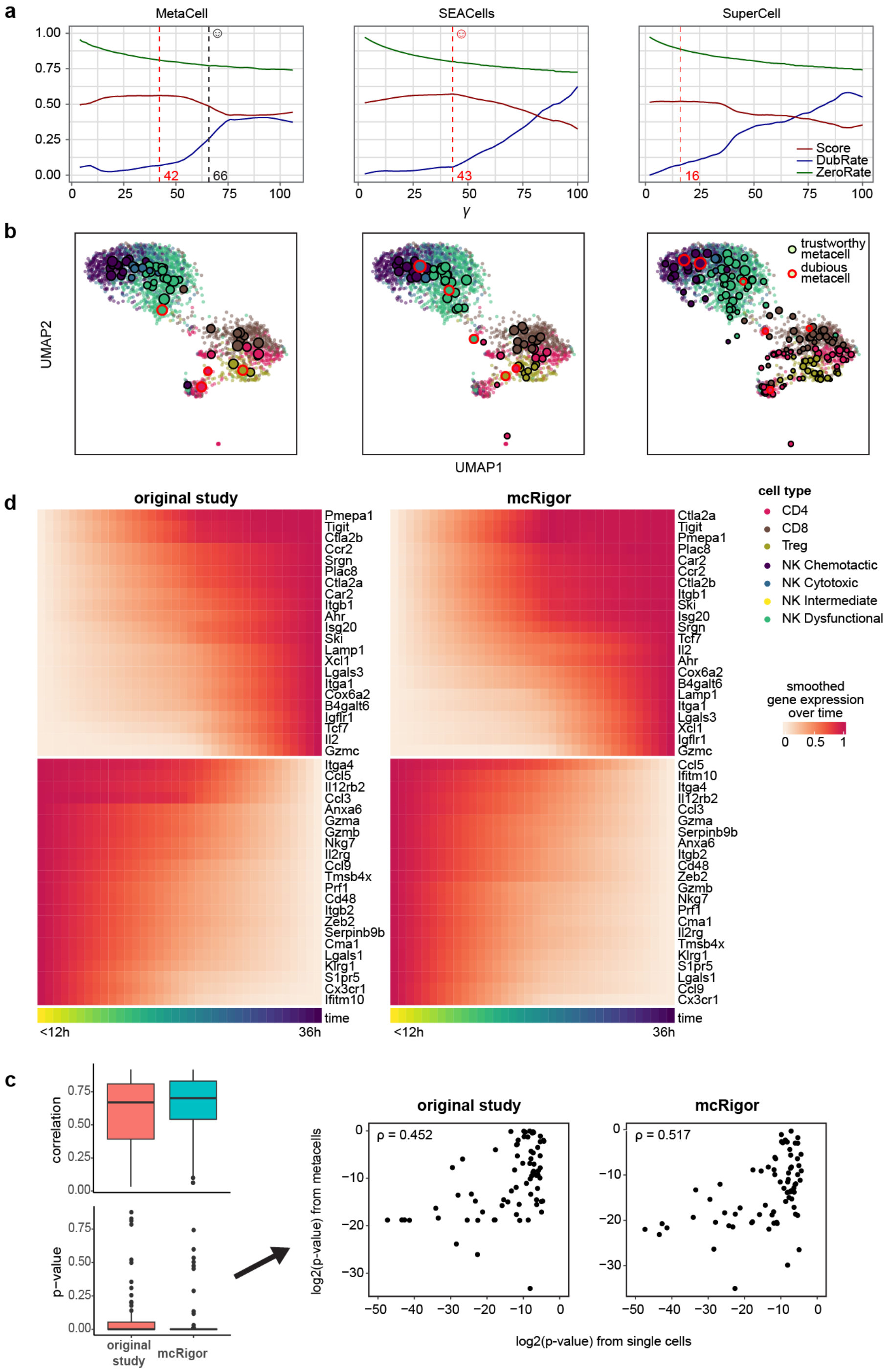

Supplementary Fig 10: Additonal results from applying mcRigor to the Zman-seq dataset. **a,** The evaluation scores provided by mcRigor. The vertical red dashed lines mark the optimal γ selected for each metacell method. The red smiling face marks the optimal metacell partition selected by mcRigor across all method-hyperparameter configurations. The black smiling face marks the metacell partition used in the original study. **b,** Single-cell UMAP plots showing the optimal metacell partitioning for each method, with dubious metacells highlighted in red circles. **c (bottom row),** The single-cell DE genes exhibited higher correlations with tumor exposure time and lower p-values when using the optimal metacell partition selected by mcRigor compared to the original partition. **d (third row),** The optimal metacell partition selected by mcRigor recovers the time-dependent genes identified in the original study.

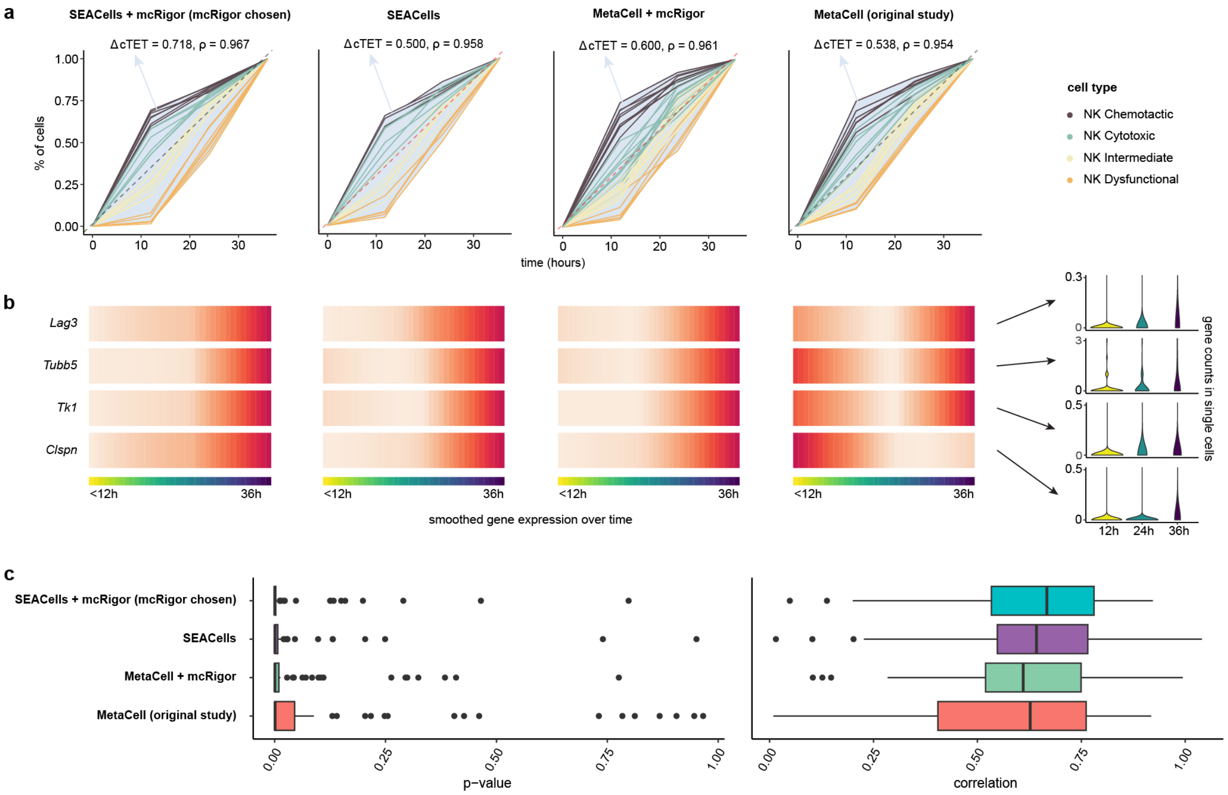

Supplementary Fig 11: Comparisons of the analysis results obtained from four different metacell partitions—SEACells + mcRigor (mcRigor chosen), SEACells, MetaCell + mcRigor, and MetaCell (original study)—for the Zman-seq dataset. a, Line plots comparing the metacells' continuous tumor exposure time (cTET) values calculated from the four metacell partitions. The value of ΔcTET, proportional to the size of the light blue area, indicates the distinction of tumor transitional stages. **b,** Smoothed gene expression profiles of four marker genes derived from the the four metacell partitions. **c,** The single-cell DE genes exhibited higher correlations with tumor exposure time and lower p-values when using the SEACells + mcRigor or MetaCell + mcRigor partition compared to the SEACells or MetaCell partition.

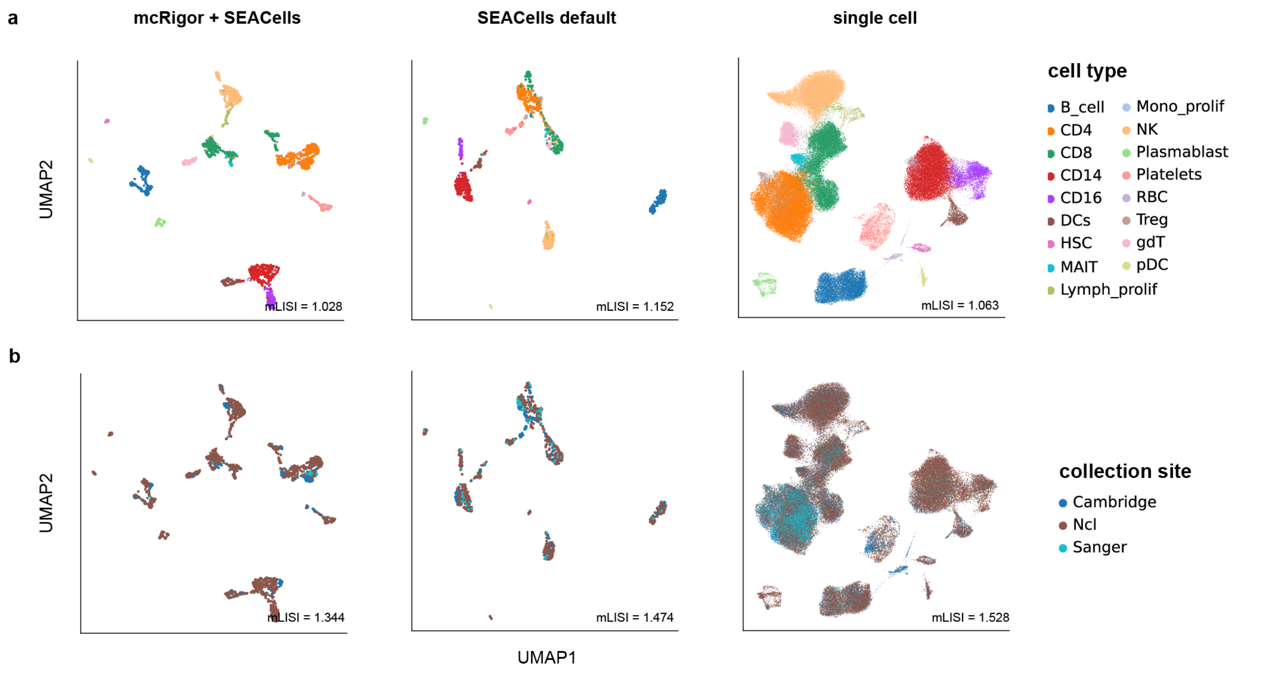

Supplementary Fig 12: mcRigor's optimized metacell partition improves integration fidelity. a, UMAP plots of integrated data after batch correction using Harmony (Korsunsky et al., 2019), showing mcRigor + SEACells metacells (left), SEACells default metacells (middle), and single cells (right), colored by cell type. Integration based on mcRigor + SEACells metacells better preserves the separation between CD4 and CD8 T cells, as reflected by a lower mLISI value based on *cell type* (1.028) compared to single cells (1.063) and SEACells default metacells (1.152). mLISI (mean Local Inverse Simpson's Index) is computed from the Harmony-corrected low-dimensional embedding; a higher mLISI indicates greater mixing across batches (e.g., cell types or collection sites), and a lower value reflects better separation. **b,** Same as (a), but with points colored by sample collection site instead of cell type. Integration based on mcRigor + SEACells metacells better preserves between-site biological differences, as indicated by the decreased mLISI value based on *collection site* (1.344), compared to single cells (1.528) and SEACells default metacells (1.474).

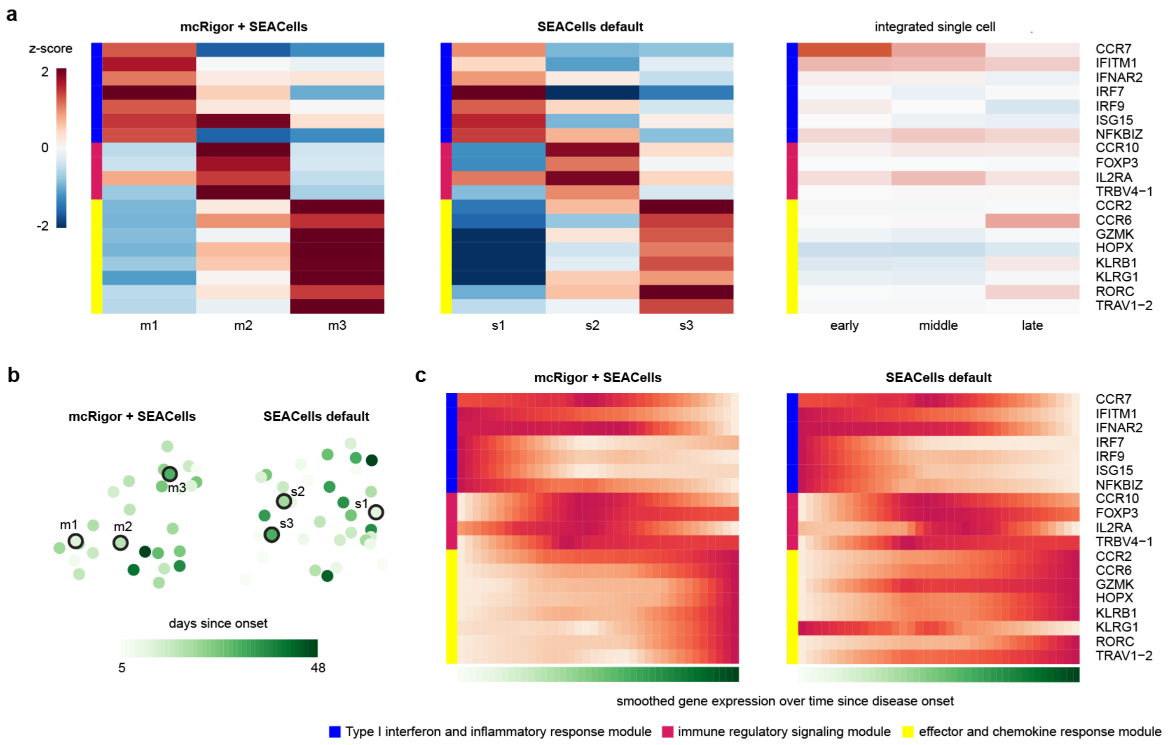

Supplementary Fig 13: mcRigor's optimized metacell partition better captures T cell response dynamics. **a,** Heatmaps showing gene expression patterns across disease stages, derived from three representative CD4 T meta2cells constructed from mcRigor + SEACells metacells (left), SEACells default metacells (middle), and pseudobulk profiles based on aggregated single cells (right), all in the Harmony-corrected embedding space. Meta2cells from mcRigor + SEACells representing early, middle, and late stages are labeled m1, m2, and m3, respectively; those from SEACells default are labeled s1, s2, and s3. **b,** UMAP plots of CD4 T meta2cells generated from mcRigor + SEACells (left) and SEACells default (right), colored by days since disease onset. In each plot, the three representative meta2cells corresponding to early, middle, and late stages are highlighted. **c,** Smoothed expression trajectories of representative immune marker genes along CD4 T meta2cells derived from mcRigor + SEACells (left) and SEACells default (right), demonstrating clearer temporal dynamics in the mcRigor-based results.

Performance of mcRigor under varying cellular heterogeneity

Cellular heterogeneity is hierarchical, spanning broad cell types, finer subtypes, and distinct cell states. To evaluate mcRigor's ability to operate across this hierarchy, we applied it to metacell partitions of the `bmcite` dataset generated by SEACells at varying granularity levels, enabling the detection of dubious metacells reflecting heterogeneity at different biological resolutions.

At a coarse granularity level ($\gamma = 90$), mcRigor successfully identified dubious metacells that mixed distinct major cell types—for example, metacell mc90-47, which included both T cells and progenitor cells (Supplementary Fig 14a). At a finer granularity level ($\gamma = 20$), it also flagged dubious metacells composed of closely related subtypes within the same major cell type—for instance, metacell mc20-9, which contained both CD8 memory T cells and CD4 naive T cells (Supplementary Fig 14b). In both cases, mcRigor exhibited high accuracy in detecting biologically impure metacells (Supplementary Fig 14c–d), demonstrating its robustness across levels of heterogeneity.

These findings support mcRigor's generalizability and effectiveness in capturing both broad and subtle forms of cellular heterogeneity.

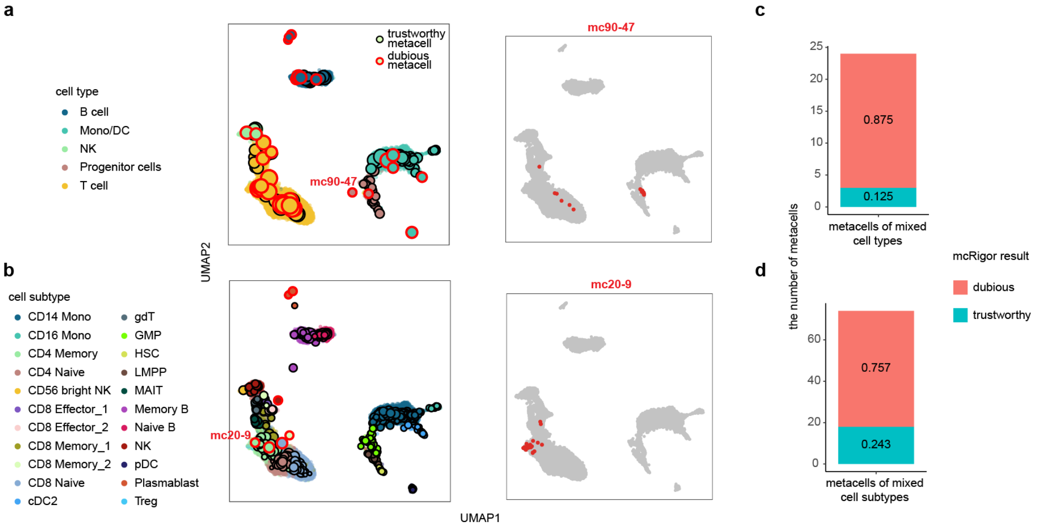

Supplementary Fig 14: mcRigor identifies dubious metacells across varying levels of cellular heterogeneity. a, Single-cell UMAP plot showing the metacell partition generated by SEACells with $\gamma = 90$ and the corresponding dubious metacells identified by mcRigor (left), along with the single-cell composition of one representative dubious metacell, mc90-47 (right). **b,** Same as **a**, but for the SEACells partition with $\gamma = 20$, highlighting another example dubious metacell, mc20-9. **c,** Bar plot showing the number of biologically heterogeneous metacells correctly identified by mcRigor as dubious versus those not flagged, in the SEACells $\gamma = 90$ partition. **d,** Same as **c**, but for the SEACells partition with $\gamma = 20$.

mcRigor two-step: an extension of mcRigor to handle dubious metacells

Following the mcRigor pipeline described in our main text, one can generate a metacell partition from a given single-cell dataset using any preferred partitioning method at a chosen granularity—either user-defined or selected via mcRigor—and then apply mcRigor to identify dubious metacells within the partition. To ensure the reliability of downstream analyses, a straightforward option is to remove these dubious metacells. However, this risks discarding cells from rare biological states, potentially

leading to the loss of critical information relevant to biological processes, such as disease occurrence. To address this issue, we propose a two-step approach, referred to as *mcRigor two-step*, which dissects the identified dubious metacells and reorganizes their constituent cells into more trustworthy metacells.

Step 1: A method–hyperparameter configuration (i.e., a metacell partitioning method with a granularity level γ_1) is either specified by the user or selected by mcRigor. This configuration is then applied to partition single cells into metacells. If mcRigor detects dubious metacells within the partition, it is re-applied to the same partition using a lower divergence score threshold. Specifically, the default threshold $q_{0.95}$ (the 95th percentile of mcDiv null values conditional on metacell size) in equation (5) is replaced with a more relaxed threshold, $q_{0.85}$ (the 85th percentile), to label more metacells as dubious for further dissection and reorganization.

Step 2: The selected metacell partitioning method is re-applied to the subset of single cells that belong to the metacells now marked as dubious. This yields a refined metacell partition under a new granularity level $\gamma_2 < \gamma_1$, which can be selected by mcRigor from the candidate set of granularity levels $2, \ldots, \gamma_1 - 1$.

We applied mcRigor two-step to two previously mentioned datasets, the COVID-19 PBMC dataset and the paired scRNA-seq and smRNA FISH dataset.

Application of mcRigor two-step on the COVID-19 PBMC dataset

We applied mcRigor two-step to the metacell partitions generated by SEACells, SuperCell, and MetaCell under $\gamma_1 = 30$ for the COVID-19 PBMC dataset (Supplementary Fig 15 and Supplementary Fig 16). Compared to the original mcRigor results, the two-step approach substantially reduced the number of dubious metacells by re-partitioning their constituent cells at a finer resolution. This reduction improved downstream analyses: the co-expression enrichment of adaptive immune response genes in COVID-19 patients—previously undetectable when using all metacells from the original partitions—became evident when using all metacells from the refined partitions generated by the two-step approach.

Specifically, starting from the SEACells partition with a first-step granularity level of $\gamma_1 = 30$, mcRigor applied a finer second-step granularity level of $\gamma_2 = 10$ to re-partition only the cells belonging to initially identified dubious metacells (11 in the healthy cohort and 13 in the COVID-19 cohort). This two-step refinement resulted in a new partition containing only 3 dubious metacells in the healthy cohort and 5 in the COVID-19 cohort (Supplementary Fig 15a, left)—a marked reduction compared to the original SEACells partition at $\gamma_1 = 30$ (Supplementary Fig 15b, left).

Using the updated SEACells partition (without filtering out the few remaining dubious metacells), we observed a statistically significant enrichment of gene co-expression within the adaptive immune response module in the COVID-19 cohort compared to the healthy cohort (p-value = 0.00158, one-sided Wilcoxon rank-sum test). This result is consistent with our earlier finding using the original SEACells partition after removing dubious metacells (11 in the healthy cohort and 13 in the COVID-19 cohort) (Supplementary Fig 15b, middle), but was not observed when using the original SEACells partition without filtering out dubious metacells (Supplementary Fig 15b, right vs. Supplementary Fig 15a, right). Moreover, after filtering out the remaining dubious metacells (3 in the healthy cohort and 5 in the COVID-19 cohort) from the updated SEACells partition, the enrichment became even more significant (p-value = 5.4e-09, one-sided Wilcoxon rank-sum test) (Supplementary Fig 15a, middle). Similar improvements in gene co-expression analysis were observed for the SuperCell and MetaCell partitions, as shown in Supplementary Fig 16a–b.

These results demonstrate that the mcRigor two-step approach improves the detection of fine-grained cell states, which are essential for capturing cell state transitions and identifying rare cell types.

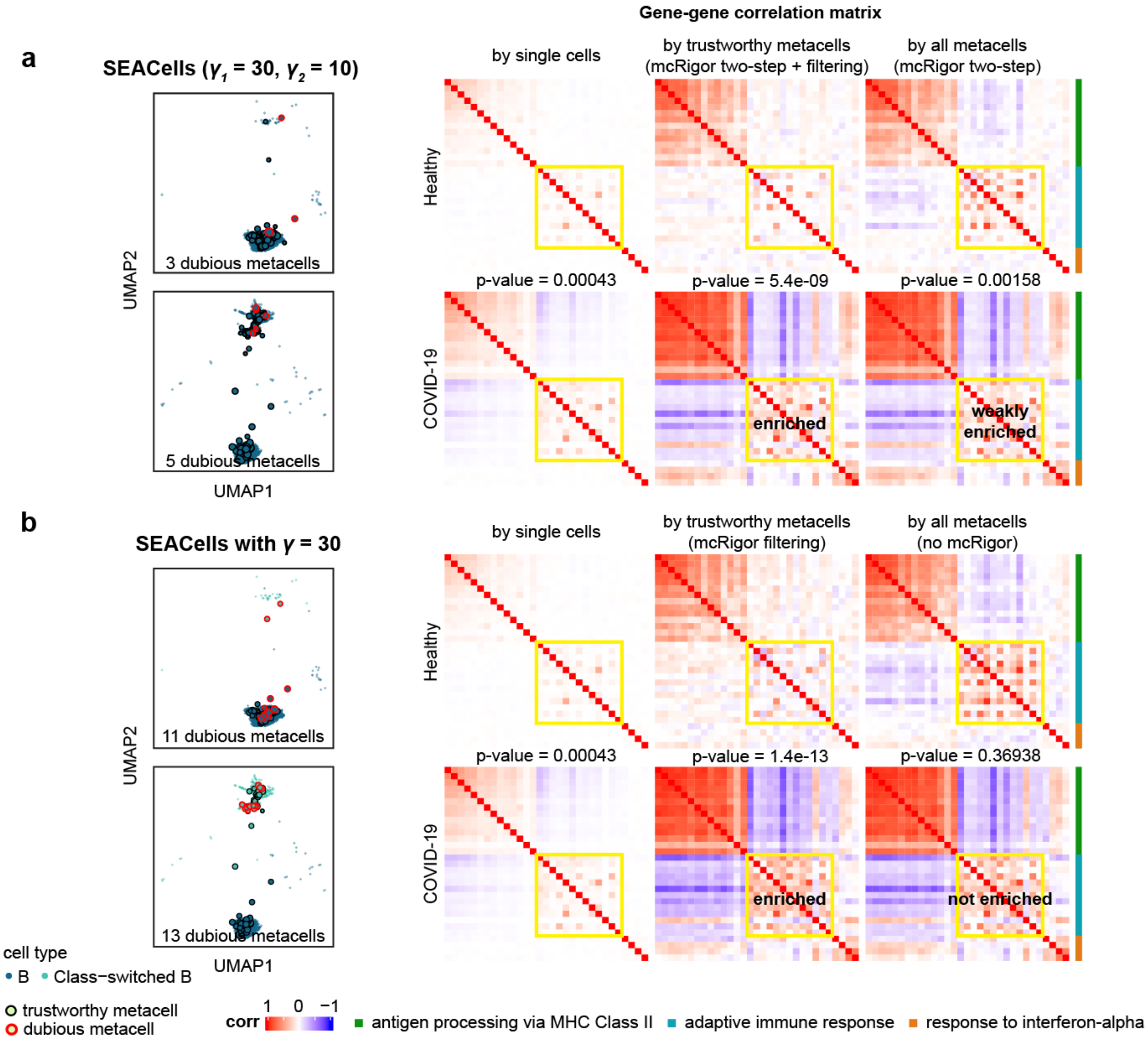

Supplementary Fig 15: mcRigor two-step reduces the number of dubious metacells and improves gene co-expression analysis in the COVID-19 PBMC dataset using SEACells ($\gamma = 30$) metacell partitions. a, Left: Single-cell UMAP plots showing metacell partitions and dubious metacells detected by mcRigor two-step under healthy (top row) and COVID-19 (bottom row) conditions. Right: Gene–gene correlation matrices for three key gene modules under healthy (top row) and COVID-19 (bottom row) conditions, based on three data types: single cells, trustworthy metacells (mcRigor two-step with filtering), and all metacells (mcRigor two-step without filtering). For each data type, the p-value comparing the correlation matrices of the adaptive immune response gene module between the two conditions was computed using a one-sided Wilcoxon rank-sum test. **b,** Same as **(a)**, but showing the results from mcRigor alone (without the mcRigor two-step extension).

Application of mcRigor two-step on the paired scRNA-seq and smRNA FISH dataset

We applied mcRigor two-step to the mcRigor-optimized metacell partitions generated by MetaCell ($\gamma_1 = 24$), SEACells ($\gamma_1 = 44$), and SuperCell ($\gamma_1 = 56$) for the paired scRNA-seq and smRNA FISH dataset (Supplementary Fig 17). Notably, mcRigor two-step reduced the number of dubious meta-

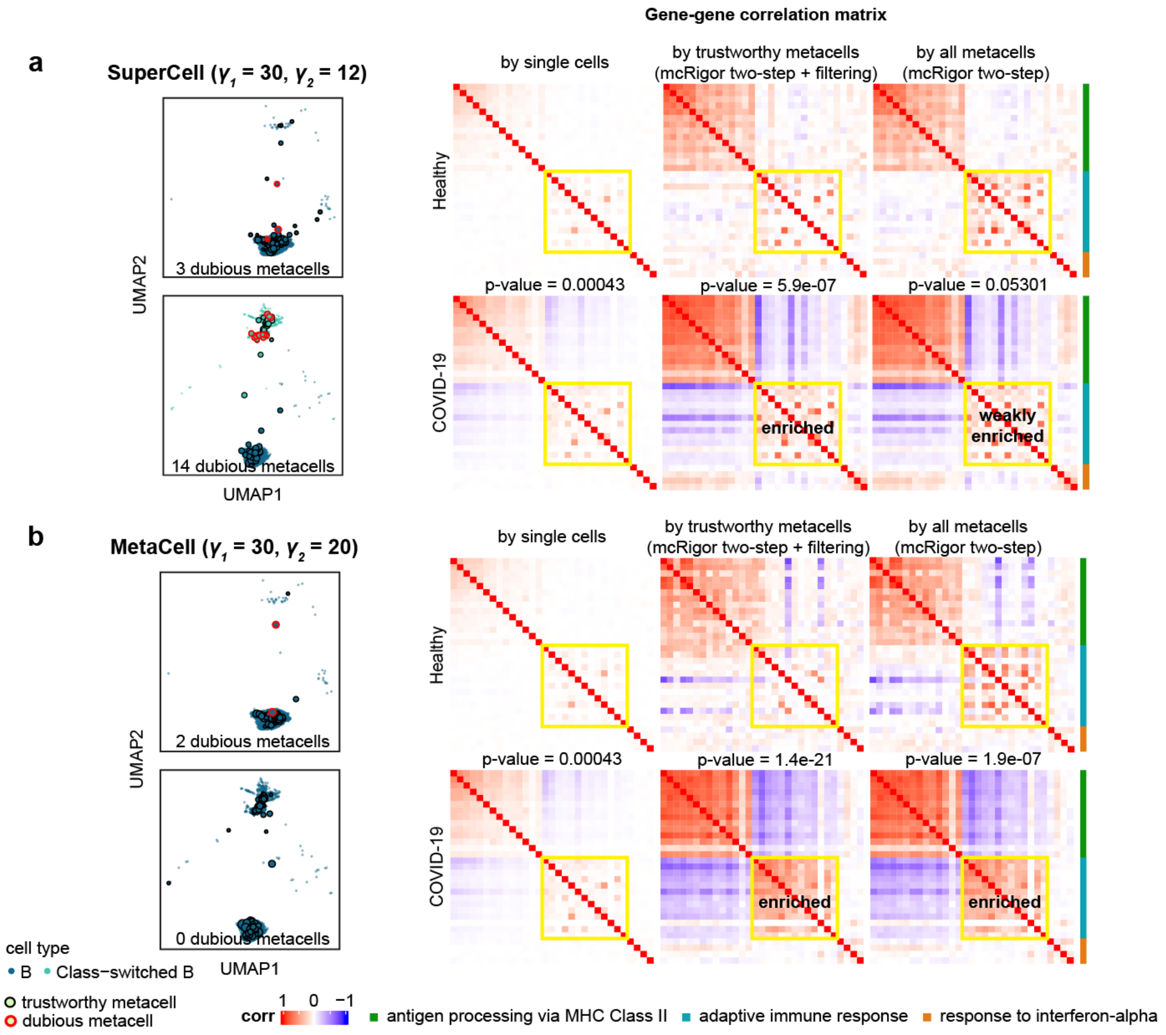

Supplementary Fig 16: mcRigor two-step reduces the number of dubious metacells and imporved gene co-expression analysis for the COVID-19 PBMC dataset based on (**a**) SuperCell partitions at $\gamma = 30$ and (**b**) MetaCell partitions at $\gamma = 30$. Left: Single-cell UMAP plots showing metacell partitions and dubious metacells detected by mcRigor two-step under healthy (top row) and COVID-19 (bottom row) conditions. Right: Gene–gene correlation matrices for three key gene modules under healthy (top row) and COVID-19 (bottom row) conditions, based on three data types: single cells, trustworthy metacells (mcRigor two-step with filtering), and all metacells (mcRigor two-step without filtering). For each data type, the p-value comparing the correlation matrices of the adaptive immune response gene module between the two conditions was computed using a one-sided Wilcoxon rank-sum test.

cells—for example, from five to three based on the MetaCell method (Supplementary Fig 17a–b)—and improved the distinction between biological and non-biological zeros. This is evidenced by better alignment between the zero proportions in the metacell-by-gene matrix and the gold-standard reference measured by smRNA FISH (Supplementary Fig 17c). For instance, under MetaCell partitioning, the proportion of zeros increased from 0.1622 (original mcRigor result) to 0.1846 after applying mcRigor two-step, approaching the smRNA FISH reference value of 0.1979. These results suggest that mcRigor

two-step more effectively resolves cell states and better distinguishes biological zeros from technical artifacts.

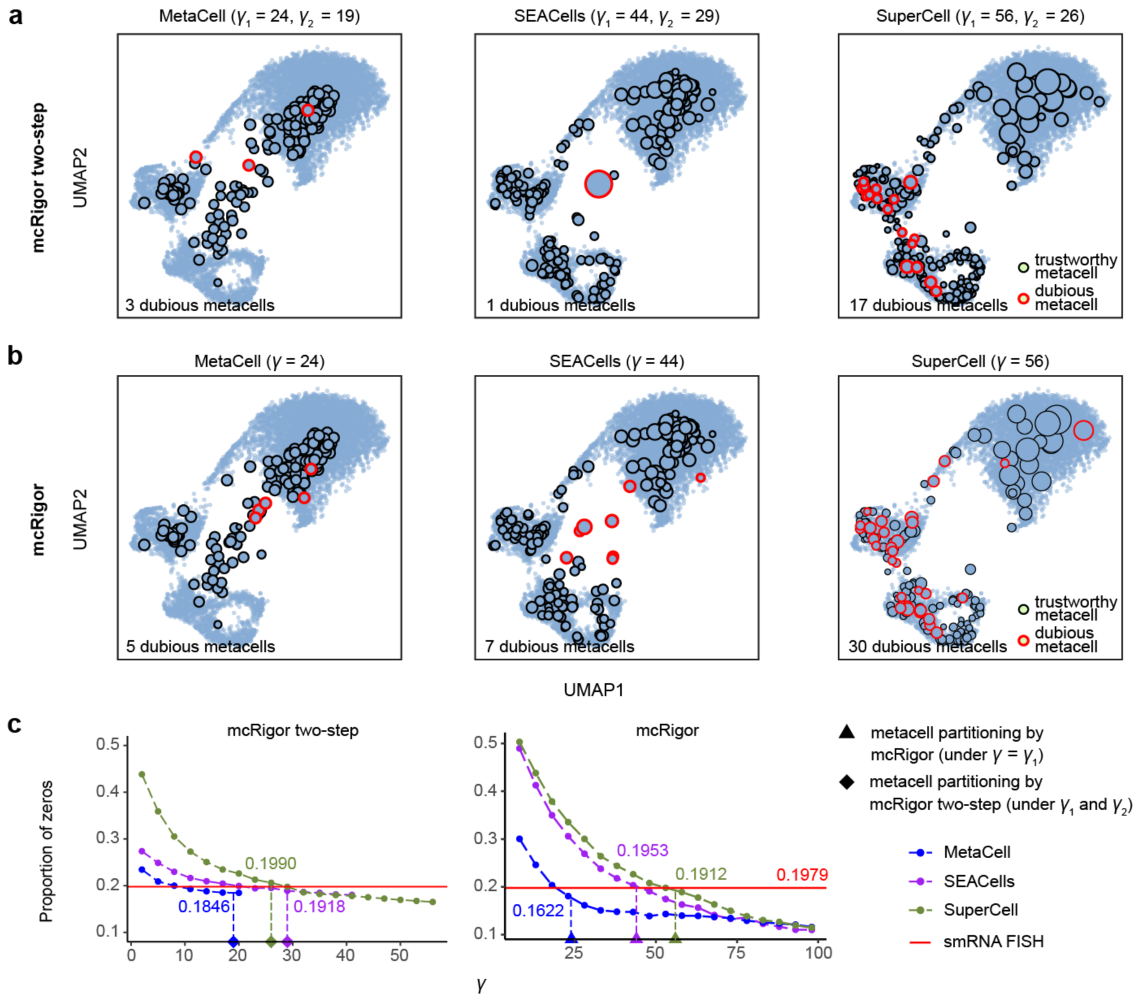

Supplementary Fig 17: mcRigor two-step reduces the number of dubious metacells and better distinguishes biological zeros from non-biological zeros in the paired scRNA-seq and smRNA FISH dataset. a, Single-cell UMAP plots showing the refined metacell partitions generated by mcRigor two-step, applied to each of the three metacell partitioning methods (MetaCell, SEACells, and SuperCell), with dubious metacells highlighted by red circles. **b,** Same as (a), but showing the original metacell partitions from the three methods, with dubious metacells detected by mcRigor (without the mcRigor two-step extension) highlighted. **c,** Line plots showing the zero proportions in metacell partitions generated by the three methods across varying granularity levels (γ for mcRigor, right; and γ_2 for mcRigor two-step, left). The refined metacell partitions from mcRigor two-step (diamonds, left) align more closely with the zero proportion observed in smRNA FISH data (red line) compared to the mcRigor-optimized partitions (triangles, right).

Capability of mcRigor two-step to resolve rare cell types

To systematically assess the ability of mcRigor two-step to capture rare cell types, we analyzed an scRNA-seq dataset of bone marrow mononuclear cells profiled by CITE-seq (the `bmcite` dataset (Stuart et al., 2019)) used in our main text, which contains two rare cell types: plasmablasts and Hematopoietic Stem Cells (HSCs), constituting 0.8% and 1.1% of the total cell population, respectively (Supplementary Fig 18a). Specifically, we first applied SEACells, SuperCell, and MetaCell with $\gamma = 50$ to generate baseline metacell partitions, and then applied mcRigor and mcRigor two-step to evaluate and refine each partition. To quantify whether a rare cell type was well captured, we examined the number and composition of trustworthy metacells in which at least 50% of the constituent single cells belonged to the rare cell type of interest (Supplementary Fig 18b-d). A rare cell type was considered well captured if it was represented by a sufficient number of such trustworthy metacells.

Notably, in the initial MetaCell partition, no trustworthy metacells representing plasmablasts or HSC cells were identified—the two plasmablasts metacells and the two HSC metacells were all marked as dubious (Supplementary Fig 18d). The original SuperCell partition contained only two very small trustworthy plasmablast metacells (the other three plasmablast metacells were marked as dubious) and none for HSCs (all nine HSC metacells were marked as dubious; Supplementary Fig 18c), failing to reliably capture either rare cell type.

After applying mcRigor two-step, selecting $\gamma_2 = 22$ for SuperCell and $\gamma_2 = 20$ for MetaCell, several trustworthy metacells representing each rare cell type emerged (Supplementary Fig 18b-c), demonstrating mcRigor two-step's effectiveness in resolving rare cell types. For the SEACells partition, which already contained trustworthy metacells for both plasmablasts and HSCs, mcRigor two-step further increased the numbers of trustworthy metacells while reducing or maintaining the numbers of dubious metacells (e.g., from 3 trustworthy and 3 dubious plasmablast metacells to 12 trustworthy and 1 dubious plasmablast metacell; Supplementary Fig 18d), confirming its utility in enhancing rare cell resolution. We also observed that some of the trustworthy HSC metacells included not only HSCs but also single cells annotated as Lympho-Myeloid Primed Progenitors (LMPPs; brown areas in the bar plots in Supplementary Fig 18b–d), which could reflect both the similarity in gene expression profiles between HSCs and LMPPs and potential inaccuracies in cell type annotation.

To evaluate the capability of mcRigor two-step to resolve even rarer cell types, we downsampled HSCs from 1.1% to 0.5% of the total cell population while retaining all other cells (Supplementary Fig 19a) and repeated the above analysis on this modified dataset. Compared to the original `bmcite` dataset, mcRigor two-step selected smaller γ_2 values for all three metacell methods on the downsampled data (Supplementary Fig 19b–d), which is intuitive since finer granularity may be required to resolve less abundant cell types. As with the original dataset, mcRigor two-step recovered trustworthy metacells representing plasmablasts and HSCs across all three methods, increasing their numbers while ensuring that the number of dubious metacells did not rise (Supplementary Fig 19b–d). However, compared to the results on the non-downsampled dataset, we observed a reduction in the number of trustworthy HSC metacells identified by mcRigor two-step, which is expected due to the smaller pool of available HSCs. These results suggest that mcRigor two-step remains effective at resolving rare cell types even at lower abundance, though its resolving power becomes increasingly challenged as the rare cell type frequency decreases. Nevertheless, mcRigor two-step offers flexibility for further extension via iterative re-partitioning of remaining dubious metacells, ultimately down to single-cell resolution if necessary. This iterative refinement provides a promising strategy for resolving even the rarest cell states without prematurely discarding them.

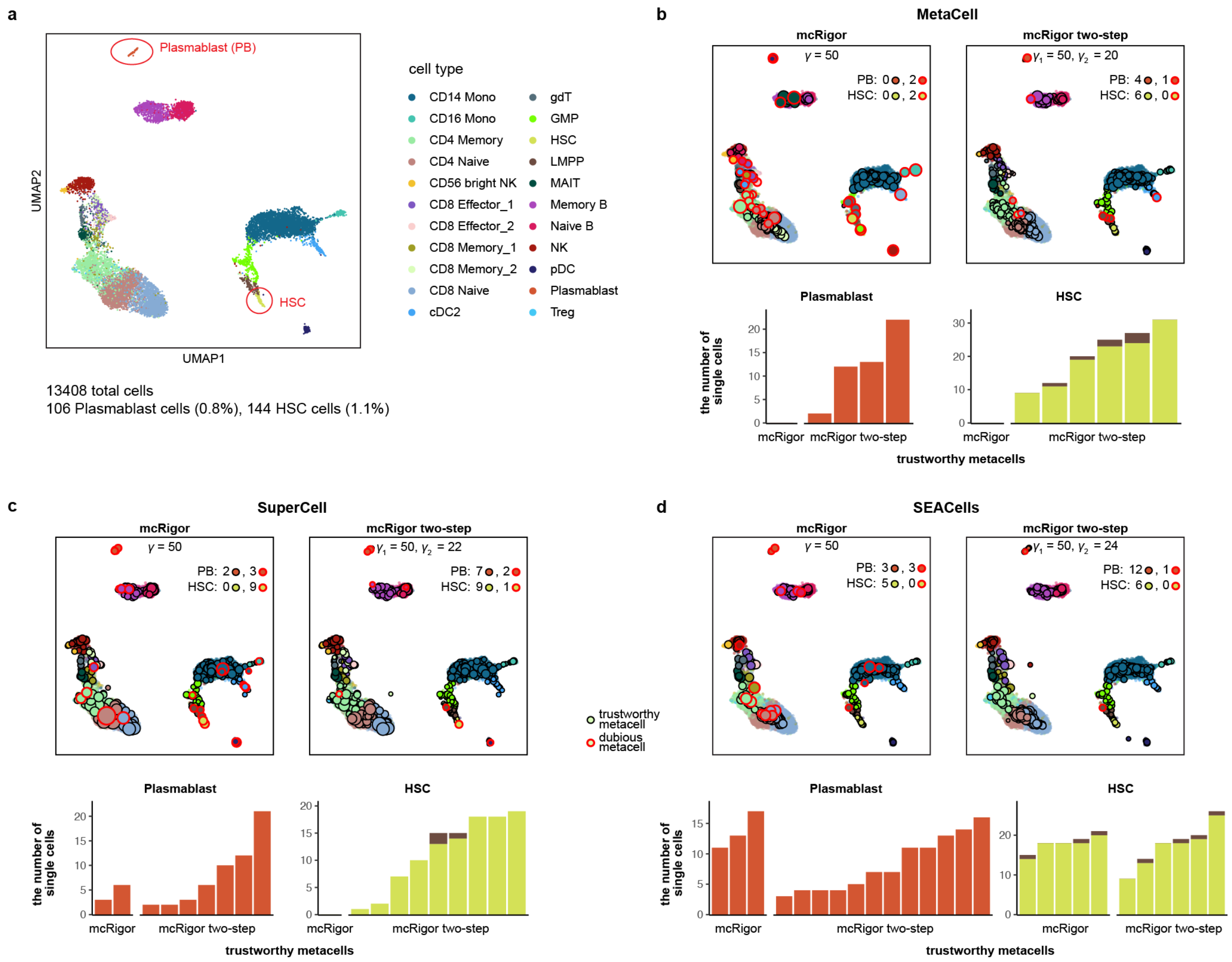

Supplementary Fig 18: Capability of *mcRigor two-step* to resolve rare cell types demonstrated using the `bmcite` dataset. a, UMAP plot of the `bmcite` dataset with the two rare cell types—plasmablasts and HSCs—highlighted. **b–d,** Demonstration of mcRigor two-step's ability to recover these rare cell types when applied to metacell partitions generated by SEACells (b), SuperCell (c), and MetaCell (d). Top: Single-cell UMAP plots showing the metacell partitions produced by the original method (left) and by mcRigor two-step (right), with dubious metacells identified by either mcRigor or mcRigor two-step highlighted in red circles. The numbers of trustworthy (black circles) and dubious metacells (red circles) representing plasmablasts and HSCs are reported at the top right of the UMAP plots (e.g., for MetaCell, mcRigor two-step detected 4 trustworthy and 1 dubious plasmablast metacells). Bottom: Bar plots showing the cell-type composition of trustworthy metacells in which at least 50% of the constituent cells belong to the corresponding rare cell type. The number of bars reflects the number of such trustworthy metacells (mcRigor: left, mcRigor two-step: right).

Distribution of metacell sizes

We observed that, at the same granularity level, metacells display substantial variability in size (Fig. 1c, Supplementary Fig. 5a, Supplementary Fig. 10b). To further investigate how metacell sizes are distributed, we plotted histograms of metacell sizes for the various cell types in the datasets we analyzed (Supplementary Fig 20, Supplementary Fig 21). Interestingly, the distributions of metacell sizes differ considerably across cell types. This variation may arise from certain biological states being more

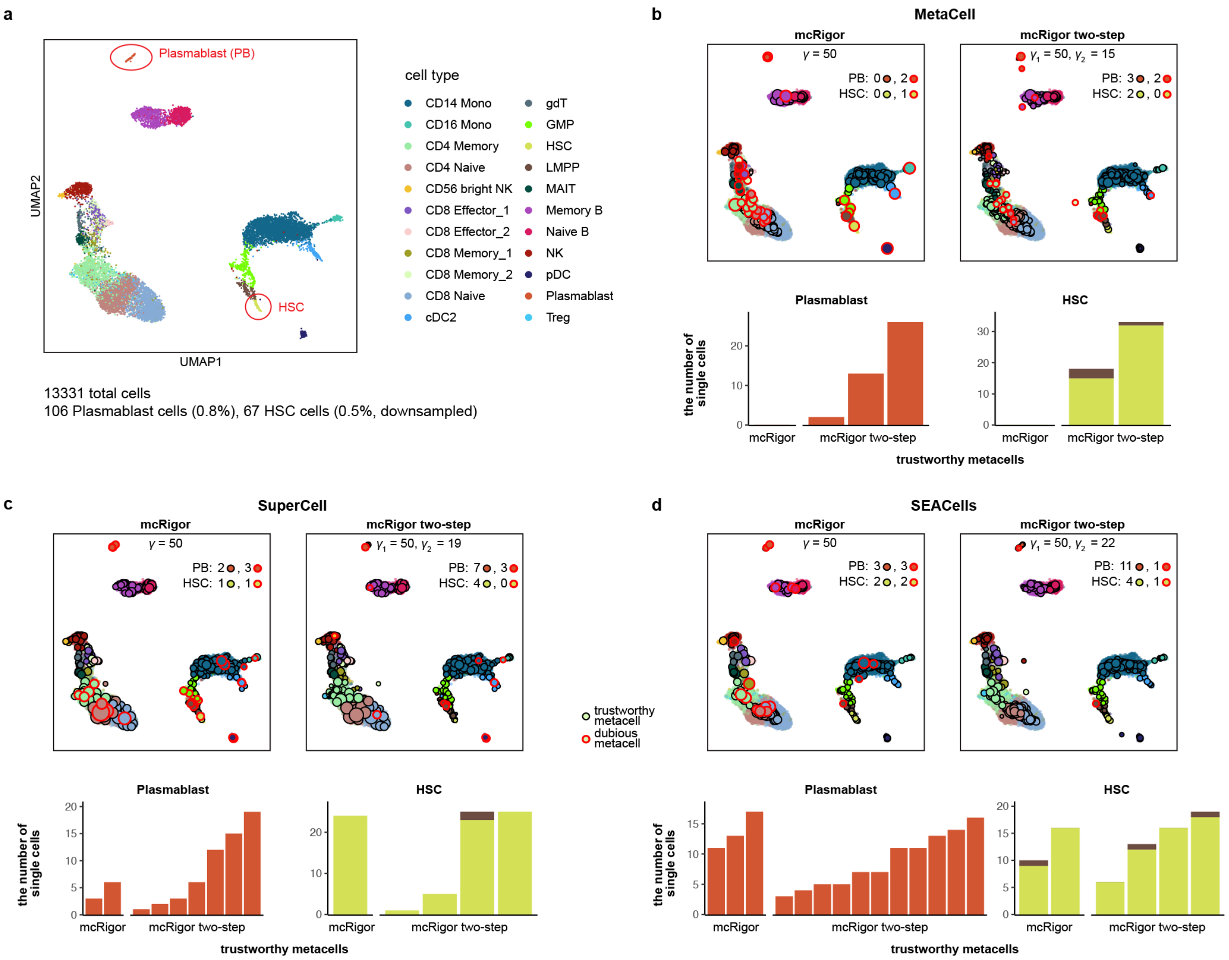

Supplementary Fig 19: Capability of mcRigor two-step to resolve rare cell types demonstrated using the `bmcite` dataset with HSC cells downsampled. a, UMAP plot of the `bmcite` dataset with the two rare cell types—plasmablasts and HSCs—highlighted. **b–d,** Demonstration of mcRigor two-step's ability to recover these rare cell types when applied to metacell partitions generated by SEACells (b), SuperCell (c), and MetaCell (d). Top: Single-cell UMAP plots showing the metacell partitions produced by the original method (left) and by mcRigor two-step (right), with dubious metacells identified by either mcRigor or mcRigor two-step highlighted in red circles. The numbers of trustworthy and dubious metacells representing plasmablasts and HSCs are reported at the top right of the UMAP plots (e.g., for MetaCell, mcRigor two-step detected 3 trustworthy and 2 dubious plasmablast metacells). Bottom: Bar plots showing the cell-type composition of trustworthy metacells in which at least 50% of the constituent cells belong to the corresponding rare cell type. The number of bars reflects the number of such trustworthy metacells (mcRigor: left, mcRigor two-step: right).

stable, thereby encompassing more cells and forming larger metacells, while less stable states include fewer cells, leading to smaller metacells. For instance, in the `bmcite` dataset, progenitor cells are generally grouped into smaller metacells, whereas T cells are aggregated into larger metacells (Supplementary Fig 21a). Furthermore, metacell size distributions vary by condition: in the COVID-19 PBMC dataset, small metacells are more abundant in the COVID-19 group than in the healthy group

(Supplementary Fig 20a), indicating less stable biological states under the diseased condition.

We also observed no clear relationship between metacell size and trustworthiness (as determined by mcRigor), indicating that dubious metacells cannot be reliably identified based solely on size.

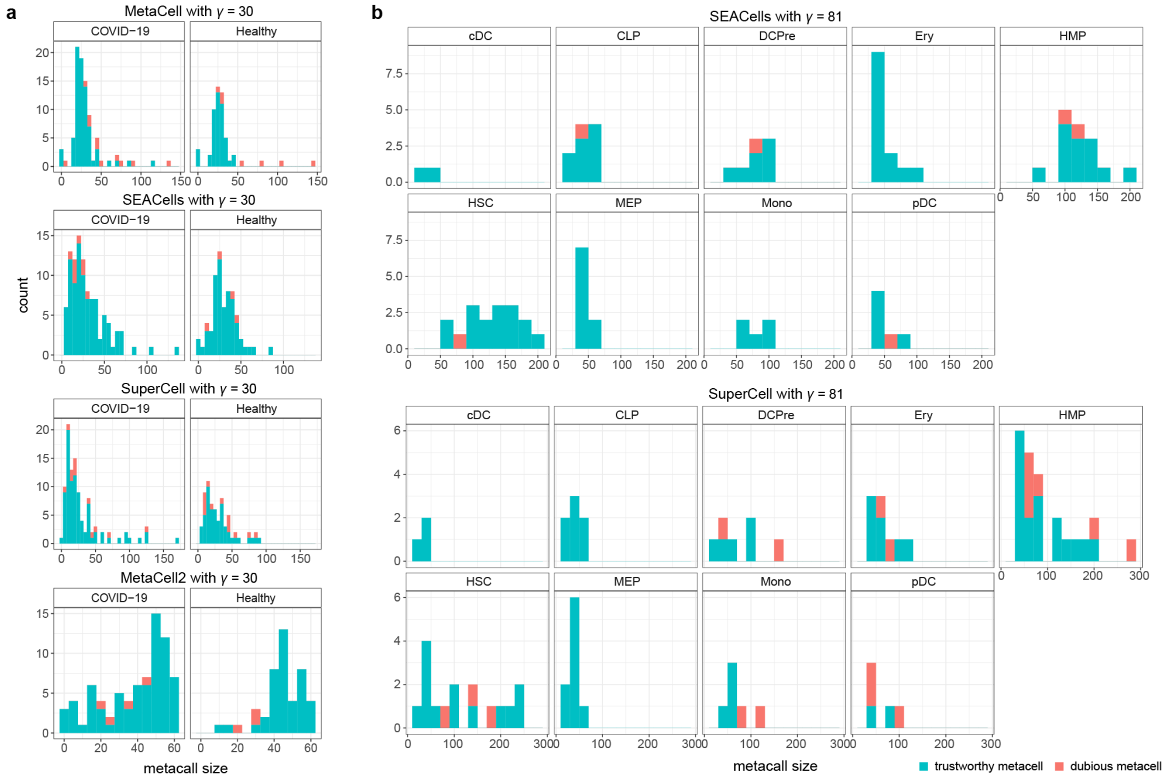

Supplementary Fig 20: Distribution of metacell sizes for (**a**) different conditions (COVID-19 versus Healthy) in the COVID-19 PBMC dataset, and (**b**) different cell types in the scMultiome dataset.

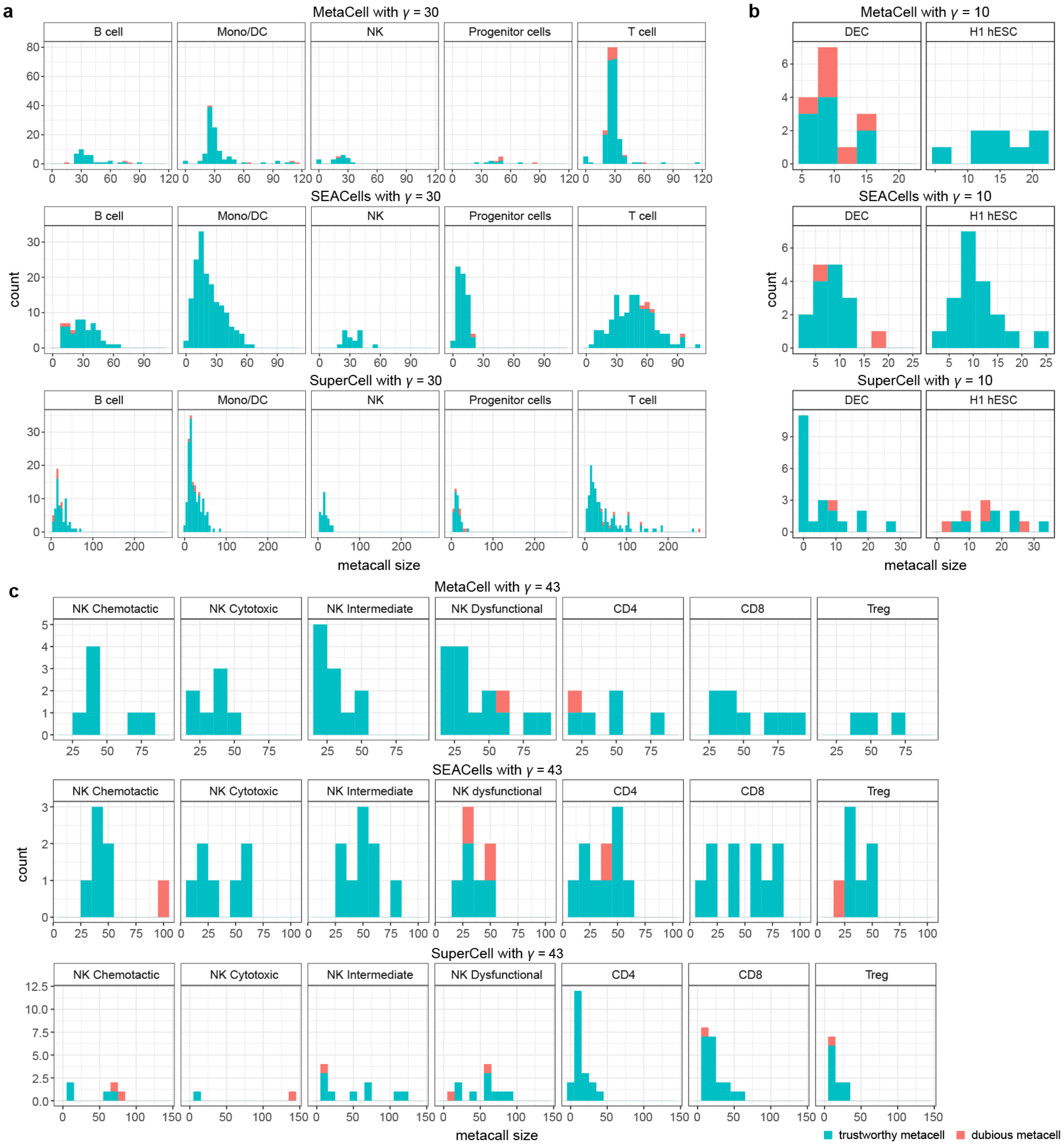

Supplementary Fig 21: Distribution of metacell sizes for different cell types in (**a**) the `bmcite` dataset, (**b**) the scRNA-seq + bulk ESC dataset, and (**c**) the Zman-seq dataset.

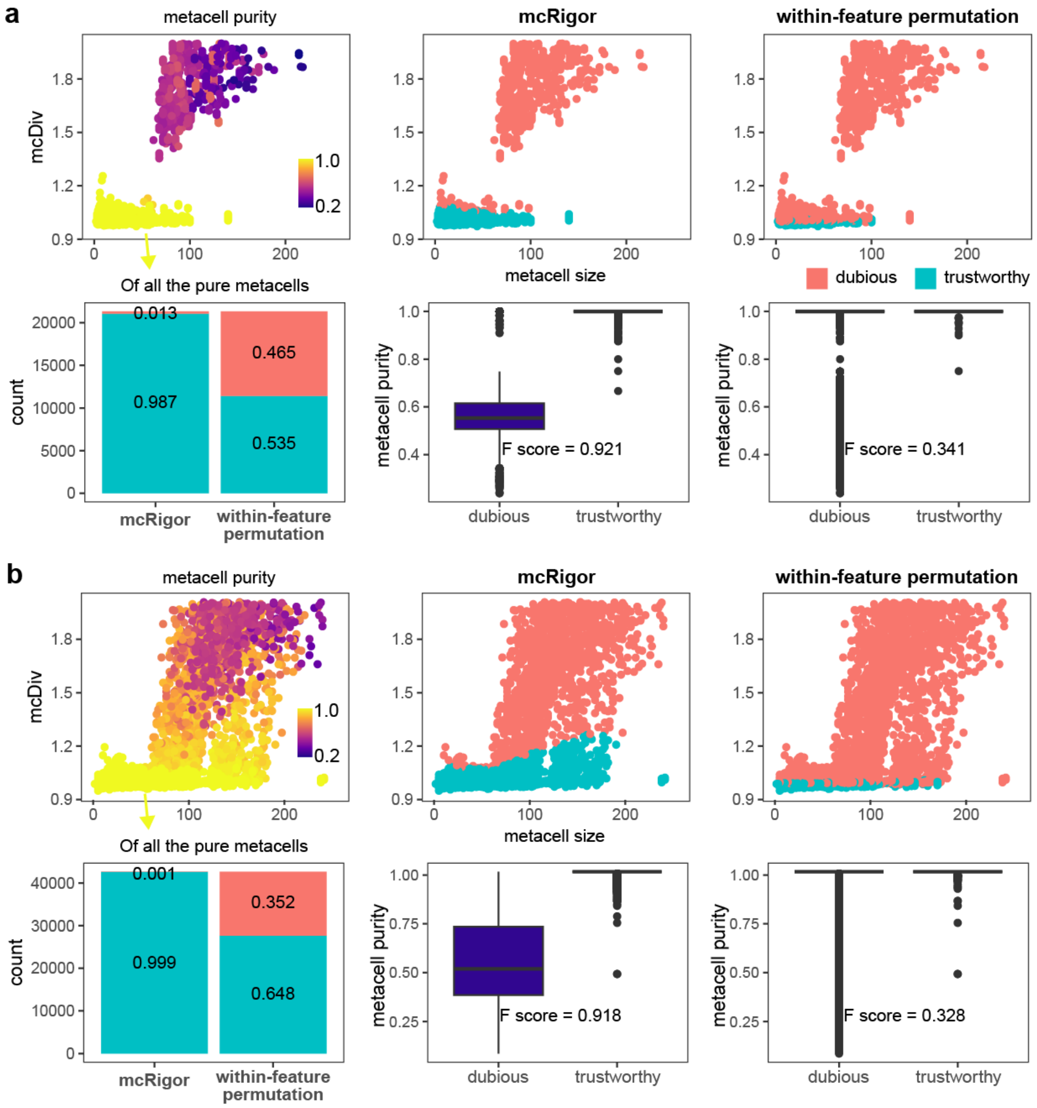

Supplementary Fig 22: Within-feature permutation alone is not a valid null for dubious metacell detection. **a,** Comparison of dubious metacell detection results obtained by applying mcRigor (double permutation) and within-feaure permutation to the metacell partition generated by MetaCell on the semi-synthetic data. **b,** Comparison of dubious metacell detection results obtained by applying mcRigor (double permutation) and within-feaure permuation to the metacell partition generated by SEACells on the semi-synthetic data.

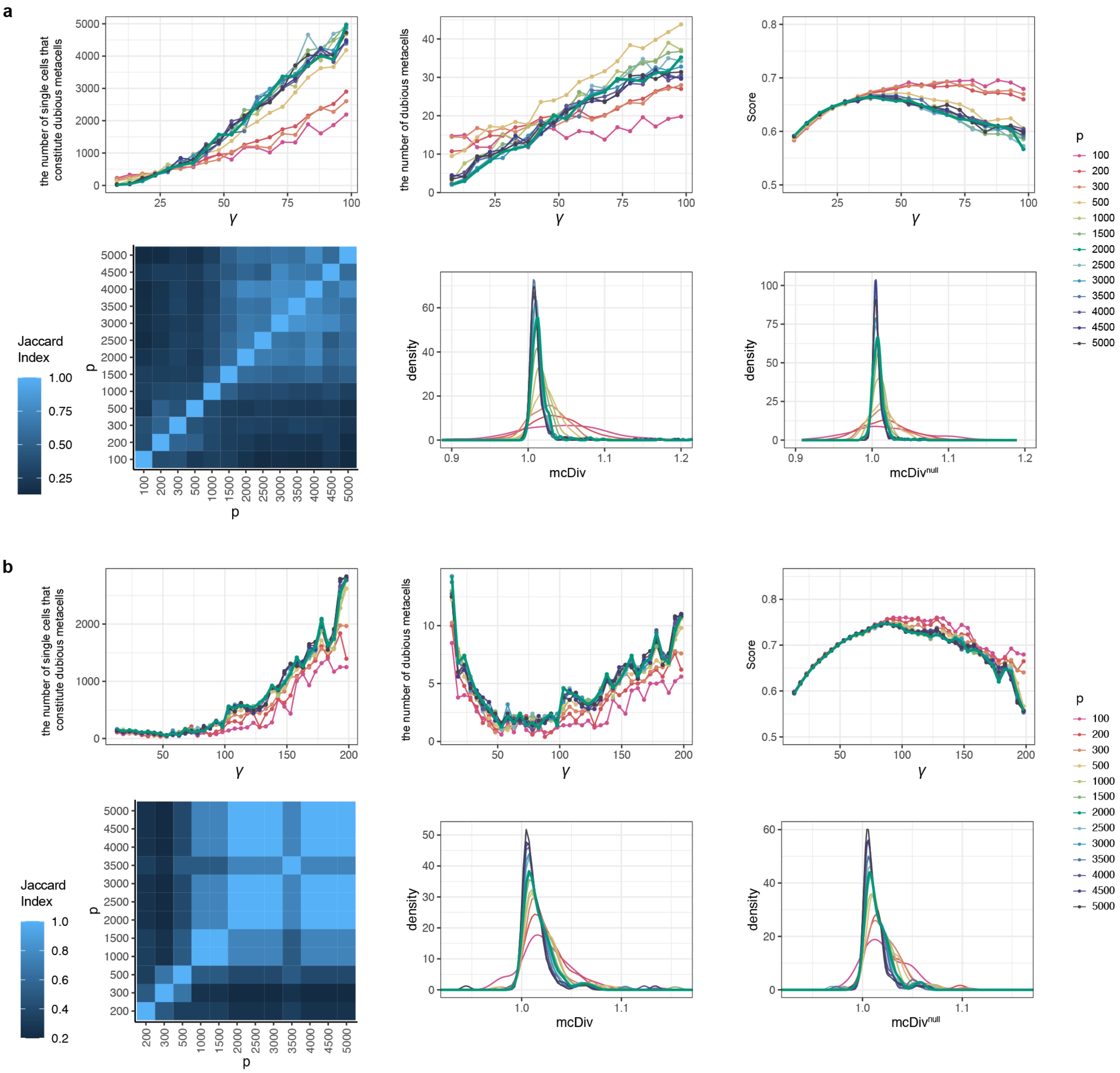

Supplementary Fig 23: Sensitivity analysis of the number of features (p) used on the `bmcite` scRNA dataset and the single-cell multiome dataset based on the SEACells method. a, Sensitivity analysis on the `bmcite` scRNA dataset by examining the number of single cells that constitute dubious metacells (top left), the number of dubious metacells (top middle), the *Score* value (top right), the distribution of mcDiv (bottom middle), the distribution of $\text{mcDiv}^{\text{null}}$ (bottom right) across different p values, and by measuring the similarity of dubious metacells found at different p values using Jaccard indices (bottom left). **b,** Sensitivity analysis on the scATAC modality of the single-cell multiome dataset using the same approaches.

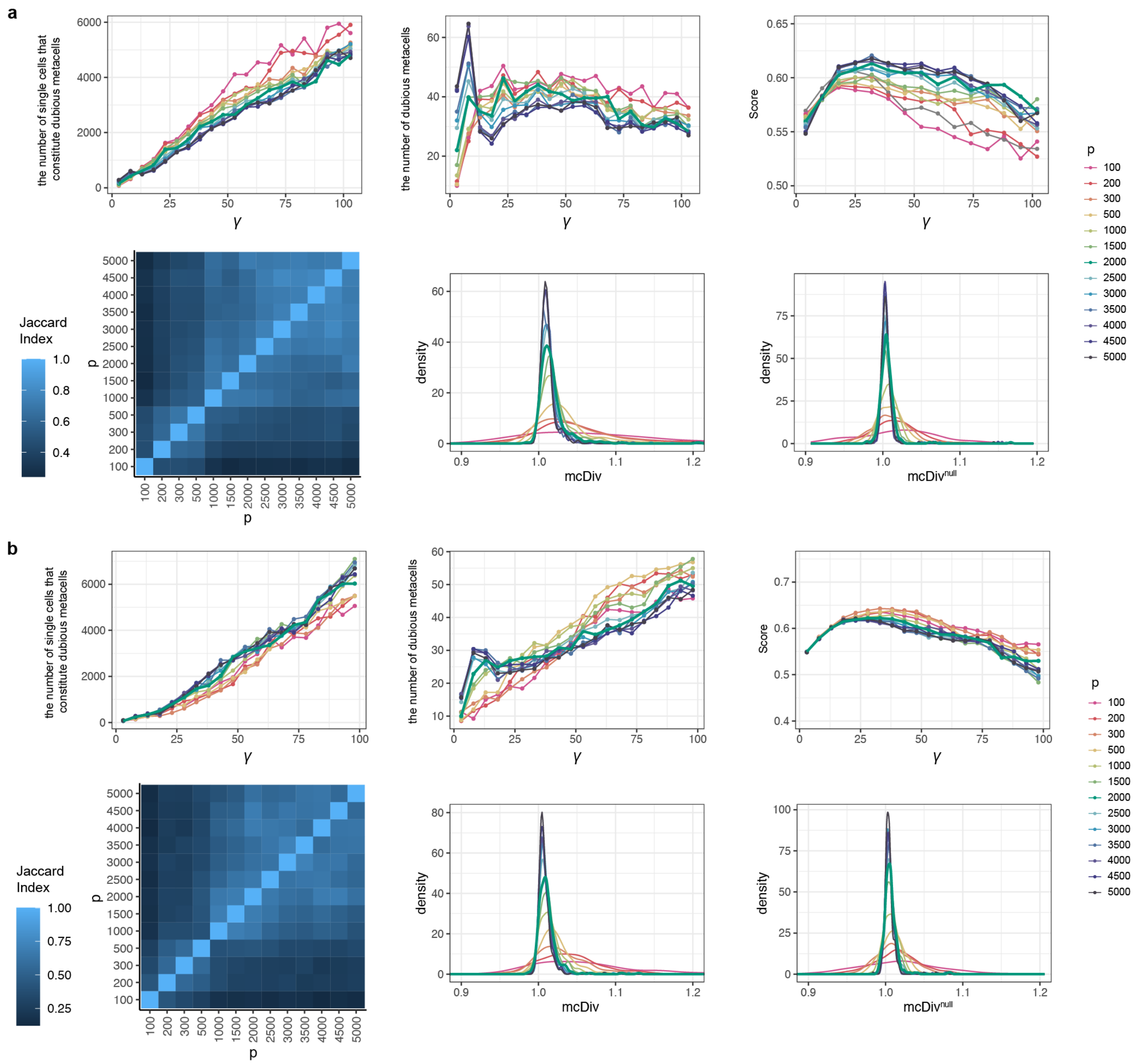

Supplementary Fig 24: Additional sensitivity analysis of the number of features (p) used on the `bmcite` scRNA dataset. a, Sensitivity analysis on the `bmcite` scRNA dataset, based on the MetaCell method, by examining the number of single cells that constitute dubious metacells (top left), the number of dubious metacells (top middle), the *Score* value (top right), the distribution of mcDiv (bottom middle), the distribution of $\text{mcDiv}^{\text{null}}$ (bottom right) across different p values, and by measuring the similarity of dubious metacells found at different p values using Jaccard indices (bottom left). **b,** Sensitivity analysis on the `bmcite` scRNA dataset based on the SuperCell method, using the same approaches.
